# Combined Effects of Obstructive Sleep Apnea and Sleep Duration on Hypertension in Korean Adults: A Nationwide Study

**DOI:** 10.3390/biomedicines13061475

**Published:** 2025-06-15

**Authors:** Seo Young Kang, Yunmi Kim

**Affiliations:** 1 Department of Family Medicine, Uijeongbu Eulji Medical Center, Eulji University School of Medicine, Uijeongbu 11759, Republic of Korea; sykang@eulji.ac.kr; 2 College of Nursing, Eulji University, Seongnam-si 13135, Republic of Korea

**Keywords:** hypertension, sleep apnea, sleep, sleep duration

## Abstract

**Background:** Obstructive sleep apnea (OSA) and abnormal sleep duration are known risk factors for hypertension. However, evidence regarding their combined effect on hypertension is limited and inconsistent. This study aimed to examine the independent and interactive associations of OSA risk and sleep duration with hypertension in Korean adults. **Methods:** We analyzed data from 14,579 adults aged ≥40 years who participated in the 2019–2022 Korea National Health and Nutrition Examination Survey. OSA risk was assessed using the STOP-Bang questionnaire and classified as low (0–2), moderate (3–4), or high (5–8). Sleep duration was self-reported and categorized as <6, 6–<7, 7–<8, 8–<9, and ≥9 h. Hypertension was defined based on measured blood pressure and antihypertensive medication use. Multivariate logistic regression was conducted to evaluate the associations. **Results:** A dose–response association was observed between OSA risk and hypertension prevalence: adjusted ORs (95 CIs) were 9.69 (8.37–11.23) for moderate and 36.58 (29.35–45.59) for high OSA risk. Sleep duration alone was not significantly associated with hypertension. However, interaction models showed a U-shaped relationship, with the lowest hypertension prevalence in those sleeping 7–<8 h. Among participants with high OSA risk, both short (<7 h) and long (≥9 h) sleep durations were associated with significantly higher hypertension risk (OR 48.49, 95% CI 19.68–119.50 for ≥9 h). **Conclusions:** OSA risk and sleep duration jointly affect hypertension risk. Individuals with high OSA risk who are short or long sleepers may require targeted interventions to improve blood pressure control.

## 1. Introduction

Hypertension is among the most critical global risk factors for mortality, as identified by the World Health Organization, and serves as a major contributor to both cardiovascular and cerebrovascular diseases [1]. Due to its typically asymptomatic nature, it is often referred to as a “silent killer”. Globally, approximately one-third of the adult population has hypertension [1]. In South Korea, the prevalence of hypertension among adults aged ≥30 years is estimated at around 33%, with 10–15% diagnosed with resistant hypertension [2].

Obstructive sleep apnea (OSA) is commonly observed in individuals with hypertension. In a study from Brazil, OSA was identified as the most prevalent secondary cause of elevated blood pressure among patients with resistant hypertension [3]. A longitudinal cohort study conducted over more than ten years in Spain demonstrated a dose–response relationship between OSA severity and the cumulative incidence of hypertension [4]. Consistent findings were reported in the Wisconsin Sleep Cohort Study in the United States, where an increased frequency of apneic events was associated with a higher risk of developing hypertension compared to individuals without sleep apnea [5]. Furthermore, in Korean data, hypertension emerged as the most prevalent comorbidity among those diagnosed with OSA [6,7]. In contrast, a longitudinal analysis based on the Victoria Sleep Cohort, which examined the association between OSA and incident hypertension in the general population, found no significant relationship between the two conditions [8].

Sleep duration has emerged as an important modifiable factor influencing blood pressure regulation. In recent decades, global sleep duration has been steadily declining, contributing to widespread sleep debt [9]. Among 35 major countries, South Korea reports the second shortest average sleep duration after Japan, raising public health concerns regarding sleep-related cardiovascular risks [10]. Insufficient sleep in individuals with hypertension activates the sympathetic nervous system during the night and into the following morning, resulting in increased blood pressure and heart rate [11]. A meta-analysis demonstrated that both short and long sleep durations are associated with elevated hypertension risk, with odds of 1.21 and 1.11, respectively [12]. In a Korean cohort study, individuals sleeping >5 h per night showed a significantly increased risk of developing hypertension; however, another study found no significant association between sleep duration and hypertension risk among women [13,14].

The growing interest in the relationship between OSA, sleep duration, and hypertension emerges from evidence suggesting that these factors not only exert independent effects on metabolic syndrome, cardiovascular disease, and cognitive function, but may also interact synergistically, resulting in a cumulative impact on blood pressure regulation [15]. Priou et al. reported that, compared to normal sleepers without OSA, the risk of hypertension was 2.51 times higher in individuals with OSA who slept ≥6 h, whereas it increased to 4.37 times in those with both OSA and short sleep duration, highlighting a potential cumulative association between these conditions [16]. Similarly, a hospital-based study in China found that, compared to individuals with an apnea–hypopnea index (AHI) <5, those with an AHI ≥5 and sleep duration <5 h had a 1.80-fold higher prevalence of hypertension, while those sleeping 5–6 h had a 1.45-fold increased risk [9]. However, findings have not been entirely consistent across populations. In a Brazilian study, OSA was associated with hypertension, but short sleep duration alone was not, and no significant interaction between OSA and short sleep duration was observed [17].

Although the association between OSA and hypertension has been consistently demonstrated across studies, findings regarding the relationship between sleep duration and hypertension have been heterogeneous. Moreover, prior research investigating the interaction between OSA and sleep duration in relation to hypertension remains limited, and the findings have been inconsistent. While a few studies have identified a synergistic effect of OSA and short sleep duration on increased hypertension risk, the potential interaction between OSA and long sleep duration, and its impact on hypertension, remains largely unexplored. Therefore, the present study aims to investigate the independent associations of OSA and sleep duration with hypertension, as well as their potential interaction, using a representative sample of South Korean adults.

## 2. Methods

### 2.1. Study Participants

**Figure 1:**
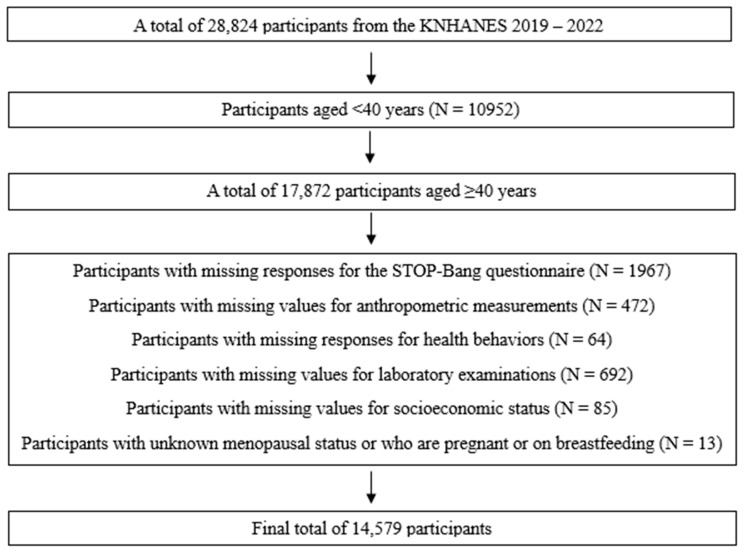
Flow of the study population.

We used data from the Korea National Health and Nutrition Examination Survey (KNHANES) 2019–2022, which contains the STOP-Bang questionnaire. The KNHANES is a nationwide cross-sectional survey conducted by the Korea Disease Control and Prevention Agency (KDCA), which applies a stratified multistage probability sampling method to represent the Korean population [18]. It consists of surveys about general health and nutritional status, health examinations, and clinical and laboratory investigations. This study was approved by the Institutional Review Board of Eulji University (EUIRB2023-073), and the need for informed consent was waived because we used the fully deidentified public database. Among the 28,824 participants in the KNHANES 2019–2022, we initially selected 17,872 participants aged ≥40 years because the STOP-Bang questionnaire was conducted on those aged ≥40 years (Figure 1). We excluded participants with missing responses for the STOP-Bang questionnaire (N = 1967), anthropometric measurements (N = 472), health behaviors (N = 64), laboratory examinations (N = 692), and socioeconomic status (N = 85), and those with unknown menopausal status or who were pregnant or breastfeeding (N = 13), leaving 14,579 participants for the final analysis.

### 2.2. Measurements of OSA Risk and Sleep Duration

The STOP-Bang questionnaire was used to evaluate the OSA risk. The STOP-Bang questionnaire is a validated and convenient tool for screening OSA and has a high concordance with the AHI obtained from polysomnography. Its usefulness as a screening tool for OSA has been also verified in the Korean population [19]. The STOP-Bang questionnaire consists of eight items, including four interview-based questions and four objective measures, each scored as “yes” (1 point) or “no” (0 point). The items are (1) snoring evaluated by the following question, “Do you snore loudly?”, (2) tiredness evaluated by the following question, “Do you often feel tired, fatigued, or sleep during daytime?”, (3) observed apnea evaluated by the following question, “Has anyone observed you stop breathing during your sleep?”, (4) high blood pressure evaluated by the following question “Do you have or are you being treated for high blood pressure?”, (5) BMI >30 kg/m^2^, (6) age >50 years, (7) neck circumference >36.3 cm, and (8) male gender. Based on sum of the scores, individuals were classified into three risk groups: low-risk (0–2 points), moderate-risk (3–4 points), and high-risk (5–8 points) [6].

Sleep duration was estimated based on responses to the survey items. In the KNHANES 2019, 2020, and 2022, the following question was used: “On average, how many hours do you sleep in a day?” In the KNHANES 2021, the following questions were used: “What time do you usually go to bed and wake up on weekdays?” and “What time do you go to bed and wake up on weekends or non-working days?” The average daily sleep duration was calculated using the following formula: (weekday sleep duration × 5 + weekend sleep duration × 2)/7. Participants were categorized into five groups according to average daily sleep duration: <6 h, ≥6 and <7 h, ≥7 and <8 h, ≥8 and <9 h, and ≥9 h [20]. To examine the combined effects of OSA risk and sleep duration on hypertension, an interaction variable was created by cross-classifying the three OSA risk groups with the five sleep duration categories, yielding 15 subgroups.

### 2.3. Measurements and Classifications of Blood Pressure

Blood pressure was measured three consecutive times in a relaxed setting on the day of the examination. The average of the second and third systolic and diastolic blood pressure readings was used for analysis. Hypertension was defined as a systolic blood pressure ≥140 mmHg, diastolic blood pressure ≥90 mmHg, or current use of antihypertensive medication for ≥20 days per month. Prehypertension was defined as a systolic blood pressure between 120 and 139 mmHg or diastolic blood pressure between 80 and 89 mmHg. Normotension was defined as systolic <120 mmHg, and diastolic was defined as <80 mmHg [21].

### 2.4. Covariates

The sociodemographic variables used in this study were age, sex, residential area (urban or rural), marital status (married/cohabitated or unmarried/separated/divorced/widowed), and education level (≤middle school graduate, high school graduate, community college graduate, or ≥college graduate). Household income was calculated as equivalent income by dividing the monthly household income by the square root of the household size and was categorized into quartiles. Occupation was classified into three categories: no occupation, manual workers, and non-manual workers [22]. High-risk drinking was defined as the intake of ≥7 standard drinks for men or ≥5 for women on a single occasion, and drinking frequency was categorized as none, <1/month, or ≥1/month [23]. Smoking status was classified into never smoker, former smoker, and current smoker. Aerobic exercise was defined as engaging in ≥150 min of moderate-intensity or ≥75 min of vigorous-intensity aerobic activity per week (yes/no). Muscle strengthening exercise was defined as performing exercises such as push-ups, sit-ups, dumbbells, weightlifting, or pull-ups on at least two days during the past week (yes/no). Laboratory variables included fasting blood glucose, glycated hemoglobin (HbA1c), total cholesterol, triglycerides, and high-density lipoprotein (HDL) cholesterol. Blood samples were collected after a minimum 8 h of fasting and were analyzed in a certified laboratory using a Hitachi Automatic Analyzer 7600 (Hitachi, Tokyo, Japan).

### 2.5. Statistical Analysis

Descriptive statistics were used to show distributions of blood pressure status according to the basic characteristics of the study participants as well as OSA risk and sleep duration. Unweighted numbers and weighted percentages were presented for categorical variables, and means and standard errors (SEs) were presented for categorical variables. To perform group comparisons, a Rao–Scott chi-square test was used for categorical variables, and analysis of variance (ANOVA) with Tukey’s post hoc test was applied for continuous variables. Multivariate logistic regression analysis was conducted to examine the independent and combined effects of OSA risk and sleep duration on hypertension. Model 1 was adjusted for age and sex, while Model 2 was additionally adjusted for sociodemographic variables (residential area, marital status, education, household income, and occupation) and health behavior variables (frequency of high-risk drinking, smoking status, and engagement in aerobic and muscle strengthening exercise). All statistical analyses were performed using SAS version 9.4 for Windows (SAS Institute Inc., Cary, NC, USA). All *p*-values were two-sided, with a significance level set at 0.05.

## 3. Results

### 3.1. Blood Pressure Status According to the Basic Characteristics of the Study Participants

Table 1 presents the distribution of blood pressure status according to the baseline characteristics of the study population. The overall prevalence of hypertension was 41.6%. Hypertension was more prevalent among older individuals, males, rural residents, and those not cohabiting with a spouse. Conversely, a higher level of education and greater household income were associated with a lower prevalence of hypertension. In terms of occupation, unemployed individuals and manual laborers exhibited a higher prevalence compared to non-manual workers. Regarding health behaviors, participants who reported high-risk alcohol consumption (≥once per month) or a history of smoking demonstrated a higher prevalence of hypertension. In contrast, engagement in aerobic or muscle-strengthening exercise was significantly associated with a lower prevalence of hypertension.

### 3.2. Blood Pressure Status According to OSA Risk and Sleep Duration

Table 2 presents the distribution of hypertension prevalence according to OSA risk level and sleep duration. Among the study population, 51.6%, 37.1%, and 11.3% were classified as having low-, moderate-, and high OSA risk, respectively. The prevalence of hypertension increased progressively across OSA risk groups: 22.6% in the low-risk group, 56.8% in the moderate-risk group, and 78.0% in the high-risk group. Regarding sleep duration, 20.0% of participants reported sleeping less than 6 h per day, while 4.7% reported sleeping 9 h or more. The lowest prevalence of hypertension (37.7%) was observed among those with a sleep duration of 7–8 h, with both shorter and longer durations associated with higher prevalence. When analyzing the 15 subgroups based on combinations of OSA risk and sleep duration, the lowest prevalence of hypertension (20.8%) was found in participants with low OSA risk and 7–8 h of sleep. In contrast, within the moderate- and high-OSA-risk groups, both short and long sleep durations were associated with substantially higher hypertension prevalence compared to the 7–8 h reference group.

**Figure 2:**
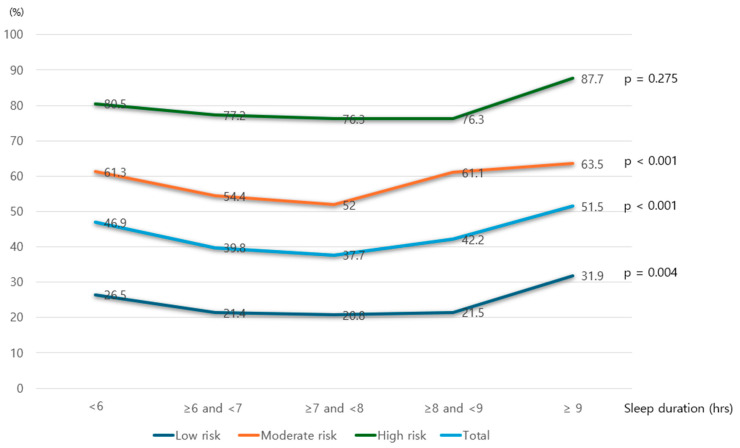
Prevalence of hypertension according to OSA risk and sleep duration.

Figure 2 illustrates the prevalence of hypertension across different categories of OSA risk and sleep duration. In all OSA risk groups, individuals with a sleep duration of 7 to 8 h exhibited the lowest prevalence of hypertension. A U-shaped association was observed, with hypertension prevalence increasing as sleep duration either decreased below or exceeded this range. Notably, across all OSA risk categories, the highest prevalence of hypertension was consistently found among individuals classified as long sleepers (≥9 h of sleep).

### 3.3. Anthropometric and Laboratory Variables According to OSA Risk

Table 3 presents the distribution of anthropometric and laboratory parameters across OSA risk categories. All measured variables differed significantly according to OSA risk level. Post hoc analyses demonstrated a consistent trend in which the values of most variables increased progressively from the low- to high-risk groups. An exception was HDL cholesterol, which exhibited an inverse trend, with lower levels observed in higher OSA risk groups.

### 3.4. Independent and Combined Effects of OSA Risk and Sleep Duration with Hypertension

Table 4 presents the results of multivariate logistic regression analyses examining the association between OSA risk, sleep duration, and hypertension. Compared to participants with low OSA risk, the ORs for hypertension were significantly higher in those with moderate (OR 9.69, 95% CI: 8.37–11.23) and high OSA risk (OR 36.58, 95% CI: 29.35–45.59). Sleep duration, when considered independently, was not significantly associated with hypertension.

When evaluating the combined effects of OSA risk and sleep duration, the reference group comprised participants with low OSA risk and a sleep duration of 7–8 h. In low OSA risk group, no significant association with hypertension was observed across sleep duration categories, except for those sleeping less than 6 h in Model 2. In contrast, among participants with moderate or high OSA risk, all sleep duration categories were significantly associated with increased odds of hypertension. Notably, in the high-OSA-risk group, the odds of hypertension were substantially elevated across all sleep durations: <6 h (OR: 35.09, 95% CI: 23.70–51.96), 7–8 h (OR: 35.38, 95% CI: 25.17–49.74), and ≥9 h (OR: 48.49, 95% CI: 19.68–119.50), with the highest risk observed among long sleepers. There was a small negative correlation between OSA and sleep duration (regression coefficient −0.0337409, *p*-value 0.028).

Model 2 was adjusted for age, sex, residence, marital status, education, household income, occupation, high-risk-drinking frequency, smoking status, aerobic exercise, muscle strengthening exercise, and body mass index.

## 4. Discussion

In this study, we investigated the independent and combined effects of OSA risk and sleep duration on hypertension using a population-based sample. Our findings revealed a clear dose–response relationship between OSA risk, as assessed by the STOP-BANG questionnaire, and the prevalence of hypertension after adjusting for potential confounders. In contrast, sleep duration alone did not show a significant independent association with hypertension. However, a significant interaction was observed between OSA risk and sleep duration. Among individuals with moderate OSA risk, sleeping <6 h was associated with an increased risk of hypertension. In those with high OSA risk, both short and long sleep durations were linked to substantially higher odds of hypertension. Notably, a typical U-shaped pattern was identified across sleep duration within each OSA risk group, with the lowest hypertension prevalence observed in those sleeping 7–8 h. This pattern persisted even after multivariable adjustment, underscoring the cumulative effect of OSA severity and aberrant sleep duration, particularly among high-risk individuals, on the likelihood of hypertension.

In this study, the STOP-Bang questionnaire was used to assess OSA risk. This tool is widely recognized as a simple yet reliable screening method for identifying individuals at risk for OSA [24,25]. One study reported that STOP-Bang scores of 0–2 as low risk, 3–4 as moderate risk, and 5–8 as high risk resulted in a specificity of 93% for the moderate-risk group and 100% for the high-risk group, while sensitivity was 47% and 37%, respectively [24]. In our study, the prevalence of high OSA risk was 11.3%, which is higher than the 7.8% reported in a systematic review of OSA prevalence based on STOP-Bang assessments, and comparable to the 12% prevalence in a recent analysis using KNHANES data [6,26]. The relatively high prevalence of OSA risk among Asian populations, including South Koreans, may be partly explained by craniofacial skeletal characteristics that differ from those of Western populations, leading to greater upper airway restriction [27,28]. Notably, the prevalence of hypertension among individuals at high OSA risk in our study was 78.0%, which is 4.7 percentage points higher than the 73.3% reported by Huh et al. [6].

Our analysis demonstrated that the likelihood of hypertension significantly increased with higher OSA risk levels compared to the low-risk group, whereas sleep duration did not show a significant effect. The association between OSA and elevated blood pressure has been consistently reported in studies conducted not only in the Korean population, but also across Western countries. A meta-analysis further supports this association, showing that the odds for hypertension were 1.18, 1.32, and 1.56 in low, moderate, and high OSA risk, respectively [29]. The mechanisms promoting hypertension in OSA are multifactorial and complex. Sympathetic nervous system activation triggered by intermittent hypoxia and/or fragmented sleep is considered the most critical pathway for blood pressure elevation in OSA [30]. In fact, the repetitive apneas and intermittent hypoxemia associated with obstructive sleep apnea activate the sympathetic nervous system, contributing to an elevated risk of diverse cardiovascular complications [31]. In patients with obstructive sleep apnea, recurrent apneic events during sleep lead to decreased blood oxygen saturation and elevated carbon dioxide levels. These physiological changes stimulate the sympathetic nervous system, resulting in increased heart rate, elevated blood pressure, and peripheral vasoconstriction. Notably, these sympathetic responses persist not only during sleep but also in wakefulness, imposing a sustained hemodynamic burden on the cardiovascular system. Consequently, the risk of hypertension, arrhythmias, heart failure, and myocardial infarction is significantly increased [32].

Additionally, OSA contributes to endothelial dysfunction, increased arterial stiffness, and fluid retention [30]. Among these, arterial stiffness plays a pivotal role in the development of resistant hypertension, which may explain the high prevalence of OSA among patients with resistant hypertension, ranging from 70% to 80% [33]. Furthermore, these relationships are bidirectional. Hypertension and arterial stiffness may also increase the OSA risk. Longstanding hypertension may impair vascular and autonomic control, which could affect upper airway tone. The prevalence of OSA among hypertensive patients is approximately 30–50% [32]. Although arterial stiffness is not a primary cause of OSA, it may exacerbate OSA severity by impairing baroreflex sensitivity, enhancing sympathetic nervous system activity, and promoting upper airway instability through hemodynamic alterations and endothelial dysfunction [34]. Moreover, OSA may increase hypertension risk by increasing plasma aldosterone level. In one study, both objective measures, such as AHI, apnea index, and lowest oxygen saturation, and subjective assessments, such as No-SAS score, of OSA were positively associated with plasma aldosterone level [35].

OSA patients with hypertension experience more severe daytime sleepiness, increased nocturia, and a higher prevalence of comorbid conditions such as diabetes, coronary heart disease, and cerebrovascular disease compared to OSA patients with normal blood pressure [36]. Regarding diabetes, one of the common comorbid conditions of OSA, more than 30% of the diabetic patients were at high risk for OSA in one study [37]. In this study, male sex, obesity-increased neck circumference, central obesity, coexisting hypertension, physical inactivity, and the presence of diabetes-related complications further increased the risk for OSA [37].

In our study, the proportion of short sleepers (defined as those sleeping <6 h per night) was 20.0%, which is substantially higher than the 13.9% reported in the 2015 KNHANES data and 15.1% in the 2010 data [38]. This finding reflects a broader trend observed in East Asian countries such as South Korea, Japan, and Singapore, where sociocultural pressures, including intense academic and occupational competition, contribute to shorter sleep durations [10,39,40]. Although our unadjusted analysis showed a U-shaped relationship between sleep duration and hypertension prevalence, with the lowest point observed among those sleeping 7–8 h, this association did not remain significant in multivariate analysis. While numerous studies and meta-analyses have explored the relationship between sleep duration and hypertension, findings remain inconsistent. A recent umbrella review concluded that longer sleep durations (≥9 h) showed “no association” with hypertension when compared to the reference of 7 h, whereas shorter sleep durations of ≤5 h and 6 h were classified as having “weak” and “suggestive” levels of evidence, respectively [41]. These discrepancies imply that the association between sleep duration and hypertension may be modulated by factors such as race, sex, age, and various health-related behaviors [41].

The relationship between the interaction of OSA and sleep duration with hypertension was first explored in a study by Priou et al., which dichotomized participants into short and normal sleepers using a 6 h cutoff. However, this study did not address the interaction between OSA and long sleep duration [16]. A more recent study conducted in China showed that, compared to primary snorers, those with OSA who slept for 5–6 h had a 45% increased odds of hypertension, and those sleeping <5 h had an 80% increased odds [9]. Interestingly, OSA patients with ≥8 h of sleep showed a reduction in the odds of hypertension. However, this study’s limitation lies in its population, as participants were patients attending a sleep clinic, which may restrict the generalizability of the findings. In our study, when evaluating combined effects of OSA risk and sleep duration using the low-OSA-risk group with 7–8 h of sleep as a reference, the odds of hypertension increased markedly in moderate- and high-OSA-risk groups when sleep duration was 6–7 h or ≤6 h. Notably, among individuals with high OSA risk and ≥9 h of sleep, the odds of hypertension increased dramatically to 48.49. These findings suggest important implications for hypertension management in patients with comorbid OSA. Standard interventions, such as continuous positive airway pressure (CPAP) and antihypertensive medications, should be considered for the management of hypertensive patients with OSA. Moreover, targeted therapeutic strategies to normalize sleep duration, particularly in high-OSA-risk individuals who are short or long sleepers, should be emphasized as a potential adjunct to blood pressure control.

This study has several limitations. First, the analysis was based on cross-sectional data, which precludes the establishment of a causal relationship between OSA, sleep duration, their interaction, and hypertension. Bidirectional associations may exist among these conditions. For instance, hypertension can negatively impact sleep quality by disrupting circadian rhythm and increasing sympathetic nervous system activity [42]. A recent meta-analysis reported that individuals with hypertension have 1.2 times higher risk for insomnia [43]. Second, OSA risk was assessed using the STOP-Bang questionnaire. While this tool has been validated as a useful screening instrument for OSA, it may not fully correspond to diagnoses confirmed by polysomnography. In general, the STOP-Bang questionnaire has high sensitivity but low specificity for detecting OSA [44]. A meta-analysis reported that the sensitivity for detecting OSA was 90%, 94%, and 96% for the cut-off using AHI ≥5, ≥15, and ≥30, respectively, whereas negative predictive value was 46%, 75%, and 90% for the cut-off using AHI ≥5, ≥15, and ≥30, respectively [45]. Due to potential false negative results, there is a limitation to using STOP-Bang questionnaire as a diagnostic tool. Furthermore, this potential misclassification introduced by using the STOP-Bang questionnaire alone might strengthen the association with hypertension. Third, the presence of individuals undergoing CPAP therapy among those at high OSA risk could not be accounted for, as the KNHANES does not collect information on CPAP usage. Individuals under treatment may have different outcomes. Fourth, other potential confounders, such as unmeasured lifestyle or health variables, were not included in the analysis. In our analysis, some ORs were extremely high, which may reflect residual confounding or sparse data bias. Fifth, sleep duration was based on self-reported questionnaire responses, which may not objectively reflect measured sleep duration such as that obtained through actigraphy. Recall bias or misclassification may exist. Lastly, the clinical or practical significance may be better evaluated using continuous outcomes, such as systolic and diastolic blood pressure. Future studies should consider these endpoints to enhance clinical relevance.

## 5. Conclusions

In conclusion, this study showed a clear dose–response relationship between OSA risk and the prevalence of hypertension. Although sleep duration alone was not independently associated with hypertension, we identified a U-shaped pattern within each OSA risk category, where both shorter and longer sleep durations were linked to higher hypertension prevalence. Notably, individuals with high OSA risk who were either short or long sleepers exhibited a markedly elevated risk of hypertension. These findings suggest that appropriate management of sleep duration in these high-risk groups is essential to reducing hypertension risk.

## Figures and Tables

**Table 1:** Distribution of blood pressure status according to the basic characteristics of the study participants.

	Total (N = 14,579)	Normotension (N = 4914)	Prehypertension (N = 3606)	Hypertension (N = 6059)	*p*-Value
Age (yrs)					
40–49	3481 (23.9)	1906 (54.8)	930 (26.7)	645 (18.5)	<0.001
50–59	3723 (25.5)	1506 (40.5)	1017 (27.3)	1200 (32.2)	
60–69	3928 (26.9)	1001 (25.5)	979 (24.9)	1948 (49.6)	
70–80	3447 (23.6)	501 (14.5)	680 (19.7)	2266 (65.7)	
Sex					
Men	6326 (43.4)	1672 (26.4)	1763 (27.9)	2891 (45.7)	<0.001
Women	8253 (56.6)	3242 (39.3)	1843 (22.3)	3168 (38.4)	
Residence					
City	11,219 (77.0)	4049 (36.1)	2759 (24.6)	4411 (39.3)	<0.001
Rural area	3360 (23.0)	865 (25.7)	847 (25.2)	1648 (49.0)	
Marrital status					
Married/cohabitated	11,287 (77.4)	3956 (35.0)	2895 (25.6)	4436 (39.3)	<0.001
Unmarried/separated/divorced/widowed	3292 (22.6)	958 (29.1)	711 (21.6)	1623 (49.3)	
Education					
≤Middle school graduate	5170 (35.5)	992 (19.2)	1122 (21.7)	3056 (59.1)	<0.001
High school graduate	4717 (32.4)	1774 (37.6)	1232 (26.1)	1711 (36.3)	
Community college graduate	1428 (9.8)	677 (47.4)	354 (24.8)	397 (27.8)	
≥College graduate	3264 (22.4)	1471 (45.1)	898 (27.5)	895 (27.4)	
Household income					
First quartile	3137 (21.5)	667 (21.3)	667 (21.3)	1803 (57.5)	<0.001
Second quartile	3644 (25.0)	1112 (30.5)	889 (24.4)	1643 (45.1)	
Third quartile	3745 (25.7)	1433 (38.3)	961 (25.7)	1351 (36.1)	
Fourth quartile	4053 (27.8)	1702 (42.0)	1089 (26.9)	1262 (31.1)	
Occupation					
No occupation	5887 (40.4)	1777 (30.2)	1292 (21.9)	2818 (47.9)	<0.001
Manual workers	3932 (27.0)	1068 (27.2)	1027 (26.1)	1837 (46.7)	
Non-manual workers	4760 (32.6)	2069 (43.5)	1287 (27.0)	1404 (29.5)	
Frequnecy of high-risk drinking					
None	9015 (61.8)	3103 (34.4)	2077 (23.0)	3835 (42.5)	<0.001
<1/month	1718 (11.8)	721 (42.0)	489 (28.5)	508 (29.6)	
≥1/month	3846 (26.4)	1090 (28.3)	1040 (27.0)	1716 (44.6)	
Smoking status					
Never smoker	8716 (59.8)	3202 (36.7)	2039 (23.4)	3475 (39.9)	<0.001
Former smoker	3701 (25.4)	1016 (27.5)	968 (26.2)	1717 (46.4)	
Current smoker	2162 (14.8)	696 (32.2)	599 (27.7)	867 (40.1)	
Aerobic exercise					
No	8861 (60.8)	2814 (31.8)	2111 (23.8)	3936 (44.4)	<0.001
Yes	5718 (39.2)	2100 (36.7)	1495 (26.1)	2123 (37.1)	
Muscle strengthening exercise					
No	11,501 (78.9)	3831 (33.3)	2786 (24.2)	4884 (42.5)	<0.001
Yes	3078 (21.1)	1083 (35.2)	820 (26.6)	1175 (38.2)	

**Table 2:** Distribution of blood pressure status according to OSA risk and sleep duration.

	Total	Normotension	Prehypertension	Hypertension	*p*-Value
OSA risk					
Low	7529 (51.6)	3697 (49.1)	2128 (28.3)	1704 (22.6)	<0.001
Moderate	5403 (37.1)	1072 (19.8)	1260 (23.3)	3071 (56.8)	
High	1647 (11.3)	145 (8.8)	218 (13.2)	1284 (78.0)	
Sleep duration (hrs)					
< 6	2917 (20.0)	831 (28.5)	719 (24.6)	1367 (46.9)	<0.001
≥6 and <7	3933 (27.0)	1395 (35.5)	971 (24.7)	1567 (39.8)	
≥7 and <8	4439 (30.4)	1607 (36.2)	1159 (26.1)	1673 (37.7)	
≥8 and <9	2607 (17.9)	892 (34.2)	615 (23.6)	1100 (42.2)	
≥9	683 (4.7)	189 (27.7)	142 (20.8)	352 (51.5)	
OSA risk and sleep duration (hrs)					
Low and <6	1403 (9.6)	596 (42.5)	435 (31.0)	372 (26.5)	<0.001
Low and ≥6 and <7	2037 (14.0)	1032 (50.7)	569 (27.9)	436 (21.4)	
Low and ≥7 and <8	2411 (16.5)	1228 (50.9)	681 (28.2)	502 (20.8)	
Low and ≥8 and <9	1358 (9.3)	702 (51.7)	364 (26.8)	292 (21.5)	
Low and ≥9	320 (2.2)	139 (43.4)	79 (24.7)	102 (31.9)	
Moderate and <6	1165 (8.0)	212 (18.2)	239 (20.5)	714 (61.3)	
Moderate and ≥6 and <7	1458 (10.0)	318 (21.8)	347 (23.8)	793 (54.4)	
Moderate and ≥7 and <8	1548 (10.6)	330 (21.3)	413 (26.7)	805 (52.0)	
Moderate and ≥8 and <9	950 (6.5)	164 (17.3)	206 (21.7)	580 (61.1)	
Moderate and ≥9	282 (1.9)	48 (17.0)	55 (19.5)	179 (63.5)	
High and <6	349 (2.4)	23 (6.6)	45 (12.9)	281 (80.5)	
High and ≥6 and <7	438 (3.0)	45 (10.3)	55 (12.6)	338 (77.2)	
High and ≥7 and <8	480 (3.3)	49 (10.2)	65 (13.5)	366 (76.3)	
High and ≥8 and <9	299 (2.1)	26 (8.7)	45 (15.1)	228 (76.3)	
High and ≥9	81 (0.6)	2 (2.5)	8 (9.9)	71 (87.7)	

OSA: obstructive sleep apnea.

**Table 3:** Distribution of the anthropometric and laboratory variables according to OSA risk.

	OSA Risk				
	Low ^a^	Moderate ^b^	High ^c^		
	Mean ± SE	Mean ± SE	Mean ± SE	*p*-Value	Post Hoc
BMI (kg/m^2^)	23.13 ± 0.04	25.01 ± 0.05	27.02 ± 0.10	<0.001	a < b < c
WC (cm)	80.62 ± 0.13	88.83 ± 0.14	94.71 ± 0.27	<0.001	a < b < c
NC (cm)	33.10 ± 0.04	36.93 ± 0.05	39.17 ± 0.07	<0.001	a < b < c
SBP (mmHg)	117.04 ± 0.24	125.22 ± 0.28	127.94 ± 0.43	<0.001	a < b < c
DBP (mmHg)	73.97 ± 0.14	78.23 ± 0.17	81.52 ± 0.32	<0.001	a < b < c
Fasting glucose (mg/dL)	99.61 ± 0.28	108.23 ± 0.43	111.29 ± 0.79	<0.001	a < b < c
HbA1c (%)	5.72 ± 0.01	5.98 ± 0.02	6.12 ± 0.03	<0.001	a < b < c
Total cholesterol (mg/dL)	198.11 ± 0.55	188.80 ± 0.69	187.41 ± 1.29	<0.001	a > b, c
Triglyceride (mg/dL)	117.24 ± 1.15	155.07 ± 2.15	178.52 ± 4.21	<0.001	a < b < c
HDL cholesterol (mg/dL)	56.56 ± 0.20	49.39 ± 0.22	46.86 ± 0.31	<0.001	a > b > c
LDL cholesterol (mg/dL)					

a: low OSA risk, b: moderate OSA risk, c: high OSA risk, OSA: obstructive sleep apnea, SE: standard error, BMI: body mass index, WC: waist circumference, NC: neck circumference, SBP: systolic blood pressure, DBP: diastolic blood pressure, HDL: high-density lipoprotein, LDL: low-density lipoprotein.

**Table 4:** Multivariate associations for independent and combined effects of OSA risk and sleep duration with hypertension.

	Model 1		Model 2	
	OR (95% CI)	*p*-Value	OR (95% CI)	*p*-Value
Independent effects				
OSA risk				
Low	1.00		1.00	
Moderate	11.63 (10.13–13.36)	<0.001	9.69 (8.37–11.23)	<0.001
High	51.55 (41.92–63.38)	<0.001	36.58 (29.35–45.59)	<0.001
Sleep duration (hrs)				
<6	1.07 (0.93–1.23)	0.339	0.96 (0.83–1.11)	0.586
≥6 and <7	1.07 (0.94–1.22)	0.284	1.04 (0.91–1.18)	0.583
≥7 and <8	1.00		1.00	
≥8 and <9	1.08 (0.93–1.26)	0.289	1.06 (0.91–1.23)	0.488
≥9	1.13 (0.90–1.41)	0.295	1.00 (0.80–1.26)	1.000
Combined effects				
OSA risk and sleep duration (hrs)				
Low and <6	0.87 (0.71–1.06)	0.172	0.76 (0.61–0.93)	0.008
Low and ≥6 and <7	0.95 (0.79–1.14)	0.553	0.93 (0.77–1.12)	0.441
Low and ≥7 and <8	1.00		1.00	
Low and ≥8 and <9	0.90 (0.74–1.10)	0.311	0.89 (0.72–1.09)	0.253
Low and ≥9	1.20 (0.88–1.65)	0.251	1.02 (0.74–1.41)	0.891
Moderate and <6	11.97 (9.49–15.10)	<0.001	9.17 (7.20–11.67)	<0.001
Moderate and ≥6 and <7	11.29 (9.02–14.12)	<0.001	8.95 (7.10–11.28)	<0.001
Moderate and ≥7 and <8	9.58 (7.79–11.77)	<0.001	7.95 (6.43–9.83)	<0.001
Moderate and ≥8 and <9	13.01 (10.20–16.60)	<0.001	10.30 (8.04–13.21)	<0.001
Moderate and ≥9	9.16 (6.61–12.71)	<0.001	7.05 (5.01–9.91)	<0.001
High and <6	55.34 (37.60–81.46)	<0.001	35.09 (23.70–51.96)	<0.001
High and ≥6 and <7	52.38 (37.70–72.77)	<0.001	35.38 (25.17–49.74)	<0.001
High and ≥7 and <8	45.17 (32.84–62.12)	<0.001	31.98 (23.02–44.43)	<0.001
High and ≥8 and <9	40.73 (27.45–60.44)	<0.001	28.74 (19.48–42.41)	<0.001
High and ≥9	79.27 (32.79–191.68)	<0.001	48.49 (19.68–119.50)	<0.001

OSA: obstructive sleep apnea, OR: odds ratio, CI: confidence interval. Model 1: adjusted for age and sex.

## Data Availability

The data presented in this study are openly available on the website of the Korea Centers for Disease Control and Prevention at https://knhanes.kdca.go.kr/knhanes/rawDataDwnld/rawDataDwnld.do (accessed on 1 August 2024).
